# The “double-edged sword” effect of cytokines in cancer: coexisting opportunities and challenges

**DOI:** 10.3389/fimmu.2025.1701405

**Published:** 2025-11-19

**Authors:** Xuan Yin, Wangzheqi Zhang, Guanhua Wang, Yawei Liu, Bing Dai, Liping Yue

**Affiliations:** 1 Department of Nursing, Institute of International Medical Science and Technology, Shanghai Sanda University, Shanghai, China; 2 Naval Medical University, Shanghai, China; 3 Anhui Medical University, Hefei, China

**Keywords:** cytokines, cancer, tumor immune microenvironment, immune checkpoint blockade, cancer therapy

## Abstract

As a key signaling molecule network in the tumor immune microenvironment, cytokines mediate intercellular communication through mechanisms such as autocrine and paracrine, exhibiting a significant “double-edged sword” effect during tumor initiation and progression. The dynamic regulation of this dual effect is influenced by the dependence on concentration, the variability within the tumor immune microenvironment, and the stages of tumor progression, ultimately representing the prolonged co-evolutionary result between tumors and the immune system. Cytokines, as a vital element of the immune microenvironment within tumors, influence cancer promotion by creating intricate networks. Therefore, disrupting this balance to alter the tumor growth environment is of great significance for achieving tumor suppression. In terms of clinical translation, the combined strategy of cytokine therapy and immune checkpoint blockade therapy has significantly improved treatment efficacy by synergistically enhancing immune activation and relieving immune suppression. Meanwhile, approaches such as monoclonal antibodies and bispecific molecules targeting pro-tumor cytokines have provided new insights for overcoming therapeutic resistance. In-depth clarification of the molecular mechanisms underlying the dual effects of cytokines, and breaking through the limitations of single targets from a network perspective, will provide a new paradigm for cancer immunotherapy from basic mechanisms to clinical applications. This will promote the upgrading of targeting strategies towards “dynamic regulation and synergistic intervention,” ultimately improving the prognosis of cancer patients.

## Graphical Abstract

Cytokines in the tumor immune microenvironment have a “double-edged sword” effect, regulated by multiple factors. Their combination with immune checkpoint blockade boosts efficacy; targeting pro-tumor cytokines aids overcoming resistance, promising better cancer immunotherapy and patient prognosis.

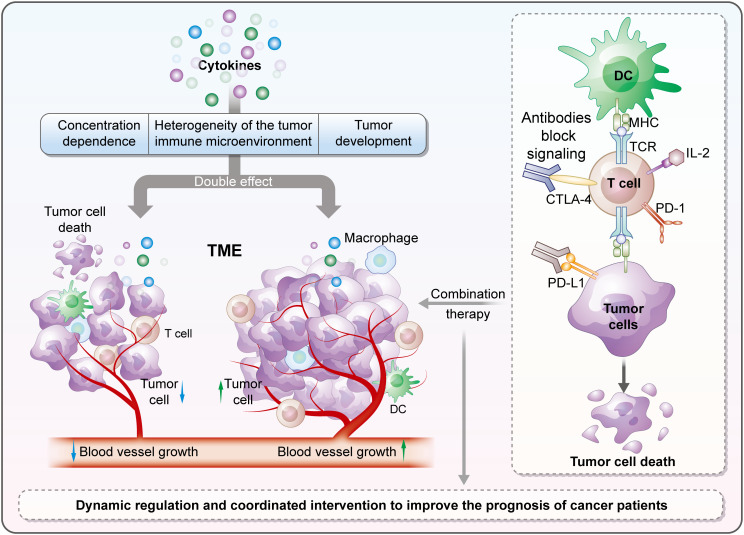

## 1 Introduction

Cytokines are protein or polypeptide substances with small sizes released by immune, tumor and stromal cells. These proteins will bind with receptors on the surface of target cells. This will release signals that will regulate biological processes in cells like growth, differentiation, and immune response. Cytokines can promote cell communication through autocrine, paracrine or endocrine mechanisms (1). Based on structure, origin, and function, cytokines can be classified into interleukins (IL), interferons (IFN), tumor necrosis factors (TNF), colony-stimulating factors (CSF), chemokines, and the transforming growth factor-β (TGF-β) superfamily, etc (2). Through these categories do not exist alone, the work of the immune system and tumors is highly interactive. Their interactions may be synergistic or antagonistic. The immune system performs homeostasis to avoid disease. Tumors, on the other hand, can initiate and progress to cause the disease. The efficiency of the immune system in fighting tumors relies greatly on the balance of their functions (3).

The tumor microenvironment (TME) is a complex ecosystem consisting of tumor cells, immune cells, stromal cells, and a cocktail of soluble factors (4). Cytokines, as key signaling molecules, profoundly impact tumor initiation, progression, and outcomes by modulating the function of immune cells, biological behavior of tumor cells, and angiogenesis (5). There is growing evidence that cytokines with anti-tumor function can also participate in facilitating tumor progression, indicating a functional duality (5). Certain cytokines can impact the fate of tumors through completely different pathways depending on the TMEs or developmental stages. The “double-edged sword”effect not only reveals the complex interaction with TME but also presents a new perspective for the development of precision immunotherapy. Recent advances in immunotherapy strategies have had a significant impact on the development of precision immunotherapy. The continuous emergence of innovative technologies such as CAR-T cell therapy and immune checkpoint inhibitors in recent years has rekindled people ‘s attention to the role of cytokines in tumor immunity (6, 7). This article, will first examine the dual mechanisms of cytokines and their therapeutic translational potential in enhancing tumor immunity.

## 2 Tumor-suppressive effects of cytokines

Cytokines like IFN-α, IFN-γ, IL-2, IL-12, IL-15 and granulocyte-macrophage colony stimulating factor (GM-CSF) have anticancer effects in tumor immunity (8). They do so by activating immune effector cells, improving antigen presentation and also acting directly on tumor cells. An important cytokine in the elimination of tumors is IFN-γ. According to research studies, Interferon gamma inhibits tumor cell growth and promotes tumor cell apoptosis. It is also known to up-regulate the expression of class I MHC on tumor cells. These mechanisms enable T cells to effectively identify and respond to tumor-associated antigens. IFN-γ can promote the activation of M1-polarized macrophages and natural killer (NK) cells, while enhancing their respective phagocytic functions and tumor-killing capabilities. Furthermore, IFN-γ can also facilitate the maturation of dendritic cells (DCs), thereby strengthening their antigen-presenting function and supporting the activation of cytotoxic T lymphocytes (CTLs) (5). IL-2 plays a critical role in driving the clonal expansion of CD4^+^ and CD8^+^ T cell populations. It not only improves the cytotoxic potential of CTLs against malignant cells but also induces the activation of NK cells and lymphokine-activated killer (LAK) cells, ultimately enhancing the ability of these cells to eliminate tumor cells (9). In contrast, IL-12 directs the differentiation of naïve T cells toward a Th1 phenotype, promotes the secretion of IFN-γ from Th1 and NK cells, and strengthens the tumor-lytic activity of CTLs and NK cells. At the same time, it limits the supply of nutrients to the tumor by suppressing angiogenesis (10). During the initial stages of tumor development, TGF-β has tumor-suppressive roles, acting by reducing cell proliferation and triggering apoptosis (11). IL-36 exerts its anti-tumor effects primarily by coordinating and activating the host’s immune system (12). Although it cannot directly stimulate effector CD8^+^ T cells, it exhibits a strong synergistic effect with cytokines such as IL-2 and IL-12. Together, they activate T cells and induce the latter to produce large amounts of IFN-γ (13). *In vivo* studies have confirmed that this immune activation can significantly inhibit the growth of tumors in models such as melanoma and fibrosarcoma. The underlying mechanism involves the induction of IL-36 receptor expression and the direct inhibition of tumor cell proliferation (14).

## 3 Tumor-promoting effects of cytokines

In contrast, during tumor progression, certain cytokines can assist tumors in evading immune attacks by promoting tumor proliferation, inducing an immunosuppressive microenvironment, and enhancing metastatic capacity. Examples include epidermal growth factor (EGF), vascular endothelial growth factor (VEGF), TGF-β, TNF-α, IL-1β, IL-6, CSF-1, chemokine (CC motif) ligand 5 (CCL5), and chemokine (CXC motif) ligand 8 (CXCL8) (15). Pro-tumor cytokines are actively involved in various phases of cancer progression, which include tumor growth, metastasis, remodeling of the extracellular matrix, evasion from the immune system, and resistance to therapies. The specific mechanisms by which cytokines promote cancer progression are provided in **Supplementary Figure 1**.

Cytokines have the ability to trigger malignant characteristics in cancer cells, including increased proliferation, migration, and angiogenesis. IL-6, produced by tumor cells as well as by macrophages and fibroblasts within the TME, facilitates the growth of tumor cells and prevents apoptosis through the activation of the Janus kinase (JAK) - signal transducer and activator of transcription (STAT) 3 signaling pathway. Moreover, IL-6 can promote the process of epithelial-mesenchymal transition (EMT) in cancer cells, enhancing their ability to invade surrounding tissues. In addition, IL-6 supports hepatic glycogen breakdown to supply energy for tumor cells and encourages the growth of vascular endothelial cells, which plays a critical role in tumor angiogenesis (16). IL-9 can directly act on hematological malignancies expressing IL-9R, including Hodgkin’s lymphoma, anaplastic large cell lymphoma, and chronic lymphocytic leukemia (17). It promotes disease progression by enhancing the survival and proliferation of tumor cells while inhibiting their apoptosis (18). Meanwhile, IL-9 also contributes to tumor development through indirect mechanisms. For example, in solid tumors such as non-small cell lung cancer and breast cancer, IL-9 can induce the immunosuppressive effects of Treg cells and mast cells in the TME, promote angiogenesis, or upregulate the expression of PD-1 molecules on cytotoxic T lymphocytes to enhance tumor immune escape (18, 19). In lung cancer metastasis models, IL-9 can further facilitate the formation of metastatic foci through Arg1 and IL-6 secreted by IL-9R^+^ stromal macrophages (20). During the progressive stage of tumors, the role of TGF-β “reverses”: on one hand, it enhances the migratory and invasive abilities of tumor cells by inducing EMT; Conversely, it creates a microenvironment that suppresses the immune response by reducing the function of effector T cells and encouraging the development of immunosuppressive cells, including Treg cells (11). Tumor cells can secrete CXCL12, which recruits these immunosuppressive cells into the TME by binding to C-X-C chemokine receptor type 4 (CXCR4) on the surface of immune cells. Meanwhile, CXCL12 can induce tumor cells to express CXCR4, promoting their metastasis to tissues with high CXCL12 expression, such as bone marrow and lymph nodes (21).

During the formation of tumor immune microenvironment (TIME), cytokines show significant tumor-promoting effects. Cytokines shape the microenvironment conducive to tumor development through a variety of mechanisms, such as driving the recruitment and phenotypic differentiation of immunosuppressive cells, inhibiting the function of anti-tumor T cells, promoting the secretion of pro-tumor cytokines, and activating related signaling pathways, thus providing support for tumor growth, progression and metastasis (22). IL-10 can inhibit the antigen-presenting function of dendritic cells (DCs) and macrophages by reducing the expression of major histocompatibility complex class II (MHC-II) molecules and co-stimulatory molecules; it suppresses type 1 helper T cell (Th1)-type immune responses by decreasing the secretion of pro-inflammatory cytokines such as IFN-γ and TNF-α; at the same time, it promotes the differentiation and longevity of Treg cells, reducing the effectiveness of effector T cells in eliminating tumor cells (23). TGF-β has the capability to diminish the cytotoxic activity of CD8^+^ T cells by lowering the levels of granzyme and perforin. Additionally, it encourages Treg cells to produce IL-10, which further amplifies the immunosuppressive impact (24). Furthermore, TGF-β can stimulate fibroblasts within the TME to release collagen, thereby creating a thick stromal barrier that obstructs the entry of immune cells into tumor tissues (25). TNF-α induces the overexpression of programmed death ligand 1 (PD-L1) in a variety of tumors, creates an immunosuppressive TME, weakens the inhibitory effect of immune checkpoints, and induces tumor cells to develop resistance to targeted therapy (18). This suggests that in the course of treatment, targeted intervention of these pro-tumor cytokines and their related pathways can affect the composition of tumor immunosuppressive microenvironment homeostasis and enhance anti-tumor immune response.

## 4 Factors influencing the dual effects

Beneath the dual effects of cytokines lies a dynamic balance of multiple regulatory factors. **Table 1** presents the roles of cytokines that exert dual effects in tumors, as well as the factors dependent on these roles. The concentration effect is one of the most prominent regulatory mechanisms: the unique and opposing roles of TNF-α in cancer depend on the cytokine concentration (29); IL-2 can enhance T cell function at appropriate levels but may induce T cell exhaustion when excessive (9). Specifically, low concentrations of TNF-α activate the NF-κB signaling pathway, inducing EMT in tumor cells, enhancing their invasiveness, and sustaining tumor-associated macrophages (30). In contrast, high-dose recombinant human TNF-α can induce hemorrhagic necrosis in syngeneic transplanted tumors in mouse models and human tumor xenografts (31). The core mechanism underlying this effect is the disruption of the tumor vascular bed, resulting in ischemic necrosis of tumor tissue (29, 31).

**Table 1:** The dual role of cytokines in tumor.

Cytokine	Anti-cancer effects	Pro-cancer effects	Dependence factors for action	References
Transforming Growth Factor-β (TGF-β)	1. Early tumor stage: Upregulates the expression of p21 protein and arrests the cell cycle progression; 2. Induces apoptosis of abnormal cells and inhibits the proliferation of tumor-initiating cells; 3. Regulates the differentiation of normal epithelial cells and maintains tissue homeostasis	1. Tumor progression stage: Activates the Smad2/3-Snail pathway, induces epithelial-mesenchymal transition (EMT), and enhances the migration and invasion capabilities of tumor cells; 2. Inhibits the cytotoxicity of CD8^+^ T cells, promotes the differentiation of regulatory T cells (Treg), and constructs an immunosuppressive microenvironment; 3. Promotes tumor angiogenesis and distant metastasis processes such as bone metastasis	Tumor development stage; Mutation status of TGF-β receptors and Smad molecules; Composition of immune cells in the TME	(11, 25–28)
Tumor Necrosis Factor-α (TNF-α)	1. High concentration: Destroys the structure of the tumor vascular bed, leading to ischemic necrosis of tumor tissue; 2. Activates the anti-tumor functions of natural killer (NK) cells and cytotoxic T lymphocytes (CTLs); 3. Induces apoptosis-related signaling pathways in tumor cells (e.g., activation of caspase-8)	1. Low concentration: Activates the nuclear factor-κB (NF-κB) pathway and induces EMT transformation of tumor cells; 2. Maintains the M2-type pro-tumor phenotype of tumor-associated macrophages (TAMs); 3. Upregulates the expression of PD-L1 in tumor cells and enhances immune escape ability; 4. Activates proliferation signaling pathways such as MAPK/Akt to promote the survival of tumor cells	Action concentration; Cell type; Signaling pathway	(29–32)
Granulocyte-Macrophage Colony-Stimulating Factor (GM-CSF)	1. In colorectal cancer: Activates the STAT1 pathway, recruits CD8^+^ T cells to the tumor microenvironment, and enhances their cytotoxicity; 2. Activates the maturation of dendritic cells and promotes cross-presentation of antigens; 3. Immunity-independent mechanism: Inhibits the clonal formation of tumor cells through autocrine signaling	1. In bladder cancer: Binds to CSF2Rα to activate the STAT3 pathway and upregulates the expression of the anti-apoptotic protein Bcl-2; 2. Promotes the accumulation of myeloid-derived suppressor cells (MDSCs) and inhibits the function of effector T cells; 3. Enhances the activity of cancer-associated fibroblasts (CAFs) and promotes matrix remodeling	Tumor type; Expression of GM-CSF receptor; Status of the immune microenvironment	(33–36)
Interferon-γ (IFN-γ)	1. Directly inhibits the proliferation of tumor cells and induces apoptosis; 2. Upregulates the expression of MHC-I molecules in tumor cells to facilitate T cell recognition; 3. Activates the anti-tumor activity of M1-type macrophages and NK cells; 4. Promotes the maturation of dendritic cells (DCs) and enhances the efficiency of antigen presentation	1. Long-term chronic stimulation: Induces the upregulation of PD-L1 expression in tumor cells and enhances immune escape; 2. Promotes the activation of Treg cells and inhibits the function of effector T cells; 3. Induces the production of drug resistance-related genes in tumor cells	Duration of stimulation; Concentration of IFN-γ; Expression level of immune checkpoint molecules in the TME	(5, 37–39)
Interleukin (IL) -2	1. Activates and expands CD8^+^ CTL cells, and enhances their tumor-killing activity; 2. Promotes the proliferation and cytotoxic function of NK cells; 3. Maintains T cell immune memory and enhances long-term anti-tumor effects	1. At low doses: Preferentially promotes the proliferation of Treg cells and inhibits the function of effector T cells; 2. Long-term use: Induces T cell exhaustion and reduces anti-tumor efficiency; 3. Promotes the activation of vascular endothelial cells and accelerates tumor angiogenesis	Administration dose; Duration of administration; Proportion of Treg cells in the TME	(9, 40, 41)
IL-6	1. Activates the STAT1 pathway and enhances the antigen-presenting function of dendritic cells (DCs); 2. Promotes the differentiation of Th1 cells and enhances adaptive anti-tumor immune responses; 3. Inhibits the self-renewal ability of cancer stem cells	1. Activates the STAT3/MAPK pathway, promotes the proliferation of tumor cells, and inhibits apoptosis; 2. Induces the EMT process and enhances the metastatic potential of tumor cells; 3. Promotes hepatic glycogenolysis to provide energy for tumor cells; 4. Accelerates the proliferation of vascular endothelial cells and promotes tumor angiogenesis	Signaling pathway; Tumor type; Expression level of IL-6 receptor	(16, 42, 43)
IL-10	1. At high doses: Enhances the proliferation and cytotoxic activity of CD8^+^ T cells; 2. Inhibits chronic inflammatory responses and reduces the occurrence of inflammation-related tumors; 3. CmAb-IL10 fusion protein: Prevents DC-mediated tumor infiltration	1. Downregulates the expression of MHC molecules on the surface of cancer cells and antigen-presenting cells, and inhibits T cell antigen recognition; 2. Induces the differentiation of Treg cells and constructs an immunosuppressive microenvironment; 3. Promotes tumor angiogenesis and enhances the invasive and metastatic capabilities of tumors	Action concentration; Tumor development stage; Combination with immune checkpoint blockers (if applicable)	(44–46)
IL-12	1. Activates the STAT4 pathway, promotes the differentiation of Th1 cells, and enhances cellular immune responses; 2. Enhances the cytotoxicity of CD8^+^ CTLs and NK cells, and promotes the secretion of IFN-γ; 3. Intratumoral injection or gene therapy: Remodels the TME and inhibits tumor growth	1. Systemic high doses: Induces severe systemic toxicity (e.g., cytokine release syndrome); 2. Long-term stimulation: Upregulates the expression of PD-L1 in tumor cells and enhances immune escape	Administration route; Dose level; Combination with PD-L1 inhibitors (if applicable)	(10, 47)

The diversity within the TME is also significant; factors like hypoxia and acidosis in the microenvironment can change the characteristics of immune cells, leading to a transformation of M1 macrophages into M2, macrophages, which subsequently modifies the cytokines they produce from pro-inflammatory to anti-inflammatory types (48). Additionally, differences in tumor development stages affect cytokine functions. For instance, TGF-β exerts a dual effect characterized by “tumor suppression in the early stage and tumor promotion in the late stage” (26, 27). In early-stage tumors, TGF-β upregulates p21 through Smad3 to inhibit the cell cycle (11). In the late stage, however, it activates Snail via Smad2/3 to induce EMT (25). The distinct effects of TGF-β on tumors at different stages depend on corresponding molecular switches, which are precisely the outcome of long-term interactions between tumors and the immune system (27).

Cytokines exhibit varying performances across different cancers. Multiple studies have shown that GM-CSF can exert either anti-tumor or pro-tumor effects (33). In colorectal cancer, GM-CSF activates the STAT 1 pathway, which recruits CD8^+^ T cells to the TME. These CD8^+^ T cells then exert cytotoxic effects against tumor cells, contributing to the anti-tumor response (34). In contrast, in bladder cancer, GM-CSF binds to CSF2Rα, activating the STAT 3 pathway. This activation promotes the expression of the anti-apoptotic protein Bcl-2 in tumor cells, enhancing their resistance to apoptosis and thereby facilitating tumor progression (35). Even more intriguing is the observed phenomenon whereby this identical cytokine can mediate opposing biological effects within the same cancer subtype (49). Acquiring a thorough and contextualized understanding of cytokine roles—whether they exert anti-tumorigenic or pro-tumorigenic functions—in a cancer-type-specific manner, alongside elucidating the molecular mechanisms that govern these dual roles, is imperative for their rational and effective application in therapeutic strategies.

## 5 Application of cytokines in targeted therapy

**Figure 1:**
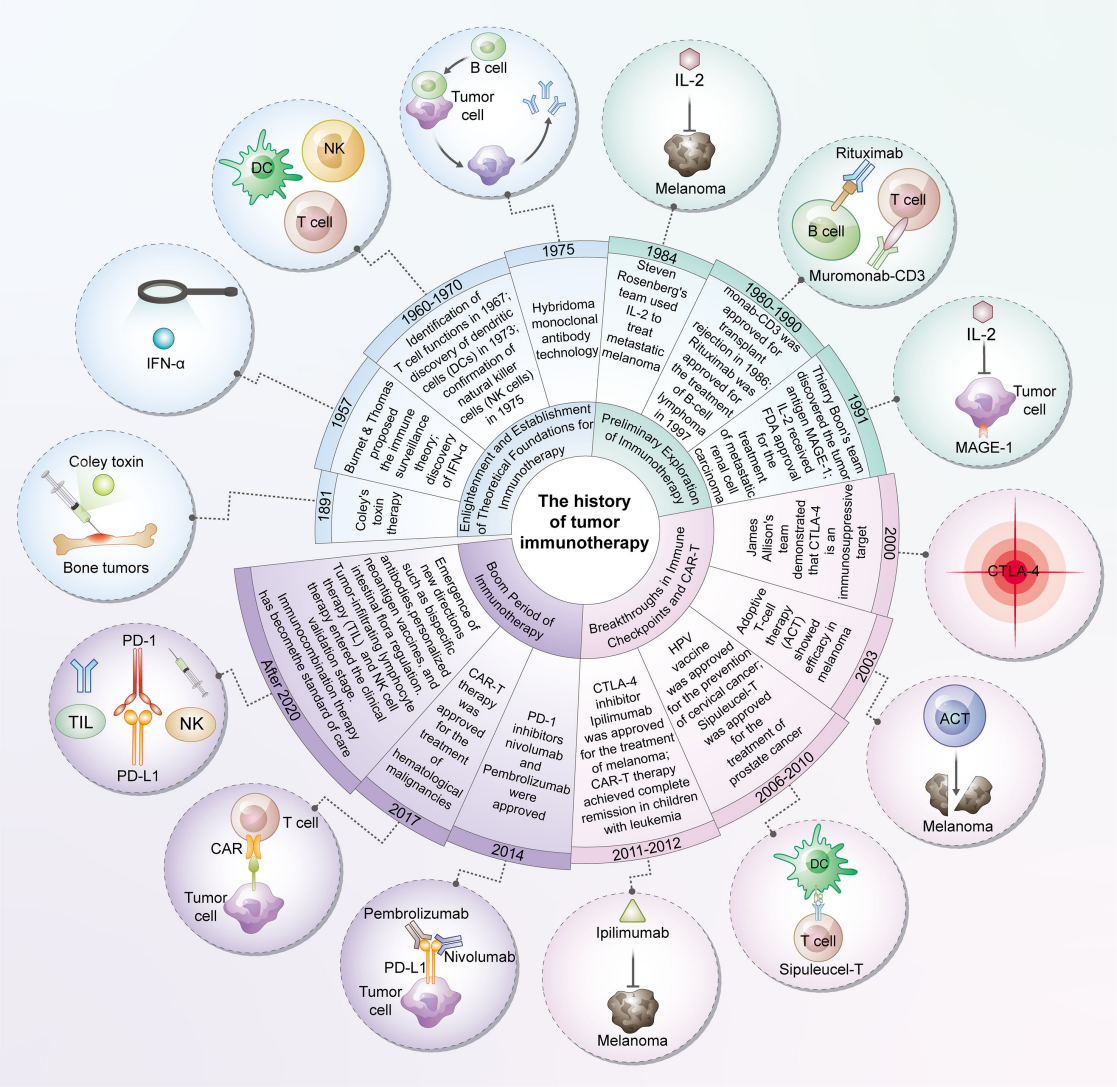
The history of tumor immunotherapy. The development of tumor immunotherapy represents a transformative history spanning from theoretical inception to clinical innovation. Originating initially from the establishment of fundamental theories such as immune surveillance, this field has gradually undergone exploratory phases including cytokine therapy and monoclonal antibody-based approaches, ultimately achieving major breakthroughs in immune checkpoint inhibitors and chimeric antigen receptor T-cell (CAR-T) therapy—breakthroughs that have revolutionized the treatment paradigm for multiple cancer types. In recent years, immunotherapy has entered a new era characterized by diversification and combined applications. Emerging directions such as personalized vaccines, bispecific antibodies, and microbial modulation continue to emerge, while immunotherapeutic combination strategies have become standard protocols in tumor treatment. Overall, tumor immunotherapy not only exemplifies the successful translation of basic scientific research into clinical practice but also demonstrates a distinct trend from the exploration of single mechanisms toward multi-targeted, personalized integrated therapy.

With the continuous advancement of cancer immunotherapy, this field has successfully achieved a revolutionary transition from theoretical establishment to clinical translation. **Figure 1** systematically summarizes the key milestones in the development of cancer immunotherapy since 1891. While cytokines function locally, their systemic use is challenged by significant toxicity and limited effectiveness. So far, only a small number of cytokines have received approval for use in treating cancer patients (50). Currently, the U.S. Food and Drug Administration (FDA) has approved IFN-α and IL-2 for the treatment of various cancers (51–54). For IFN-α, in the adjuvant treatment of melanoma, it has been shown to increase the overall survival from 2.8 years to 3.8 years. Additionally, the proportion of patients with persistent disease-free survival has been improved by 42% (52). Notwithstanding the drawbacks associated with these therapies, including significant side effects and the requirement for high dosages, pertinent clinical research has shown the effectiveness of cytokines in enhancing patient outcomes (2).

In clinical settings, alternative immunotherapies, especially immune checkpoint blockade (ICB) therapy, have predominantly supplanted these cytokines, owing to their enhanced effectiveness and improved safety profile (55, 56). Immune checkpoints constitute a group of molecules within the immune system that normally serve to modulate the strength and duration of immune responses, thereby preventing the excessive activation of immune cells that could harm healthy tissues. The typical representatives of immune checkpoint molecules include cytotoxic T lymphocyte-associated antigen 4 (CTLA-4), programmed death receptor 1 (PD-1) and PD-L1 (57). Within the TME, cancer cells often use these immune checkpoint molecules: by binding to the corresponding receptors on the surface of immune cells, they transmit inhibitory signals, resulting in T cells and other immune cells unable to effectively identify and attack cancer cells, thus helping tumors to evade immune surveillance and clearance (58). Immune checkpoint blockade therapy uses drugs such as specific antibodies to block the interaction between immune checkpoint molecules and their ligands, reversing this immunosuppressive state. For example, antibodies that target PD-1/PD-L1 obstruct the interaction between PD-1 and PD-L1, whereas antibodies against CTLA-4 impede the activity of CTLA-4, thereby enabling T cells to restore their functions and improve their capacity to identify and destroy cancer cells, leading to tumor management and eradication (59). At present, ICB therapy has demonstrated notable effectiveness across several cancers, including melanoma, non-small cell lung carcinoma, and renal cell carcinoma, positioning it as a key strategy in cancer immunotherapy (6). However, this therapy also has certain limitations: some patients may experience immune-related adverse reactions, not all patients can benefit from it, and there are issues such as intrinsic tumor resistance. Therefore, its clinical application is still under continuous exploration and optimization (60, 61).

The prospect of integrating cytokines with various immunotherapeutic approaches, in conjunction with progress in drug delivery systems and protein engineering, has rekindled enthusiasm for the use of cytokines in the treatment of cancer (62). In order to overcome the drug resistance mechanism of ICB monotherapy, one of the key directions of current clinical research is to combine cytokine therapy with ICB. The evaluation of the combined application of ICB with cytokines such as IFN-α and IL-12 in a number of trials has shown that such combined treatment regimens can synergistically enhance anti-tumor immunity (63). In general, cytokines represent promising yet complex targets within the TIME. Cytokines have the ability to enhance the infiltration of immune cells and facilitate the partial activation of lymphocytes; therefore, therapies that utilize cytokines might aid in overcoming both primary and acquired resistance to ICB, thereby maximizing clinical advantages across a diverse array of patients (64). Clinical data have demonstrated that the combination of IFN-α-1b and PD-1 monoclonal antibody exhibits favorable anti-tumor activity and acceptable toxicity in Chinese patients with metastatic melanoma, including those with cutaneous, acral, and mucosal subtypes (65). A latest study revealed that for microsatellite-stable colorectal cancer liver metastases—a typical type of “cold tumor”—the combined use of LIGHT cytokine and anti-CTLA-4 antibody can synergistically remodel the TME (66). Specifically, LIGHT activates T cells, while the anti-CTLA-4 antibody reverses the exhaustion state of activated T cells and eliminates inhibitory immune cells, thereby achieving effective control of tumor growth (66).

In addition, the advancement of monoclonal antibodies and receptor inhibitors aimed at pro-tumor cytokines like VEGF, IL-6, and TGF-β represents a significant achievement within cancer therapy. These cytokines, which support various dimensions of cancer progression, facilitate processes such as tumor expansion, metastasis, remodeling of the extracellular matrix, immune system avoidance, and resilience against treatment (15). By neutralizing these cancer-facilitating cytokines or inhibiting their receptors, the efficacy of cancer immunotherapy could potentially be improved. Presently, multiple approaches to inhibit these cytokines have been established, which include neutralizing antibodies, bispecific antibodies, small-molecule inhibitors, cytokine traps, small interfering RNA (siRNA), and peptides (2). Certain cytokine antagonists, including anti-TGF-β and anti-VEGF antibodies, have demonstrated considerable promise in boosting the effectiveness of different immunotherapy modalities, including ICB, while addressing treatment resistance (67).

Cytokine-centered therapies for cancer encounter two main obstacles: significant toxicity and less than ideal efficacy. The negative effects linked to cytokine treatment, including capillary leak syndrome seen with high doses of IL-2, highlight the need for approaches that can minimize toxicity without compromising effectiveness (68). Additionally, tumor heterogeneity and the complexity of the TME result in variable patient responses to these treatments, highlighting the importance of identifying biomarkers to predict treatment responses and guide therapeutic choices (63). For cytokine-based cancer therapies to be successfully translated into clinical practice, improvements are required in the following areas: optimizing pharmacokinetic and pharmacodynamic properties; refining local delivery strategies; gaining a deeper understanding of environment-dependent interactions within the TME; and optimizing combination therapies (69). Moreover, since cytokines typically act synergistically with other cytokines and chemokines to form regulatory loops, targeting multiple cytokines simultaneously may yield more effective anti-tumor effects than single-agent cytokine-targeted drugs (42). It is important to highlight that a majority of cytokines serve multiple functions and have unique effects at various phases of tumor progression. Consequently, the knowledge of the precise role that cytokines play in each patient is vital for the improvement of therapies targeting these molecules.

## 6 Discussion

The interplay between tumor cells and the immune system over a long period of time is basically responsible for the dual role of cytokines in tumor immunity. This can not only illustrate the complexity of tumor immune regulation, but also provide a critical breakthrough for breaking the therapeutic bottleneck. Scale inhibition of tumor development related with certain cytokines convinces investigators in a study to optimize their use in tumor treatments. Conventional therapies utilizing cytokines have not produced great results due to inevitable side effects. The next-generation targeting strategy is now shifting towards “precision regulation”: using monoclonal antibodies to block the functionality of protumor factors. Alongside this method, a genetic engineering approach will also be employed to modify cytokines and enhance their anti- tumor specificity and reduce the immunosuppressive effect. Cytokine-targeted treatment combined with immune checkpoint inhibitors can relieve immune suppression as well as enhance immune activation. As they possess complementary mechanisms of action, they can form a synergistic effect.

However, the implementation of these strategies still faces numerous challenges: How to precisely target cytokines in the TME without disrupting systemic immune homeostasis? How to develop personalized cytokine regulation protocols based on individual patient differences? To address these issues, we need to further decipher the dynamic change rules of cytokine networks and develop more precise intervention methods. In future research, only by deeply understanding the dual roles of cytokines and their dynamic variation patterns can we achieve precise regulation of tumor immune responses. Cytokine-targeted therapy, based on theoretical mechanisms and continuously integrated with other treatment modalities, is expected to expand its therapeutic potential, improve its clinical application, and ultimately enhance the prognosis of cancer patients.

Although combination therapy can partially overcome the limitations of single-target therapy, the dynamic adaptability of the cytokine network in the TME can still lead to treatment resistance in patients. Therefore, the treatment strategy needs to shift from “target intervention” to “network regulation”. The “Network equilibrium Hypothesis” holds that the cytokine network in the TME maintains a stable pro-tumor state through complex interactions and feedback loops. Breaking this balanced state rather than merely targeting individual cytokines can disrupt the tumor’s adaptability to the microenvironment, restore the anti-tumor immune response, and ultimately achieve effective tumor suppression. This shift from focusing on individual targets to regulating the entire network reflects a deeper understanding of the complexity of the TME and provides a new direction for developing more effective cancer immunotherapy strategies.

## Supplementary material

Supplementary Figure 1 Mechanisms of cytokines promoting cancer progression. Cytokines can promote the proliferation and survival of tumor cells; facilitate epithelial-mesenchymal transition (EMT) and stemness maintenance of tumor cells, thereby fostering a more invasive phenotype; drive angiogenesis; participate in the activation of fibroblasts into cancer-associated fibroblasts (CAFs) and the remodeling of the extracellular matrix (ECM), which in turn promote tumor immune evasion and therapeutic resistance; and pro-inflammatory cytokines induce dysregulated inflammation to support tumor initiation and progression. Additionally, cytokines act on immune cells within the tumor microenvironment to form an immunosuppressive tumor microenvironment, helping tumors evade immune surveillance and further proliferate.

## Glossary

CAR-T: Chimeric Antigen Receptor T-cell
CAF: Cancer-associated fibroblast
CCL5: Chemokine (CC motif) ligand 5
CSF: Colony-stimulating factor
CTLA-4: Cytotoxic T-lymphocyte-associated antigen 4
CTL: Cytotoxic T lymphocyte
CXCL8: Chemokine (CXC motif) ligand 8
CXCL12: Chemokine (CXC motif) ligand 12
CXCR4: C-X-C chemokine receptor type 4
CXCR7: C-X-C chemokine receptor type 7
DC: Dendritic cell
ECM: Extracellular matrix
EMT: Epithelial-mesenchymal transition
EGF: Epidermal growth factor
FDA: U.S. Food and Drug Administration
GM-CSF: Granulocyte-macrophage colony-stimulating factor
ICB: Immune checkpoint blockade
IFN: Interferon
IL: Interleukin
JAK: Janus kinase
LAK: Lymphokine-activated killer
MAPK: Mitogen-activated protein kinase
MHC: Major histocompatibility complex
M-CSF: Macrophage Colony-Stimulating Factor
NF-κB: Nuclear factor-κB
NK: Natural killer
PD-1: Programmed death-1
PD-L1: Programmed death-ligand 1
PI3K/Akt: Phosphatidylinositol 3-kinase/protein kinase B
SDF-1: Stromal cell-derived factor-1
siRNA: Small interfering RNA
STAT: Signal transducer and activator of transcription
TAM: Tumor-associated macrophage
TGF-β: Transforming growth factor-β
Th1: Type 1 helper T cell
TIME: Tumor immune microenvironment
TME: Tumor microenvironment
TNF: Tumor necrosis factor
TRAIL: Tumor Necrosis Factor-Related Apoptosis-Inducing Ligand
Treg: Regulatory T
VEGF: Vascular endothelial growth factor.
