# Supplementary figures and images for: The “double-edged sword” effect of cytokines in cancer: coexisting opportunities and challenges
Source: Front Immunol. 2025 Nov 19;16:1701405. doi: 10.3389/fimmu.2025.1701405 (PMC12672462; doi:10.3389/fimmu.2025.1701405)

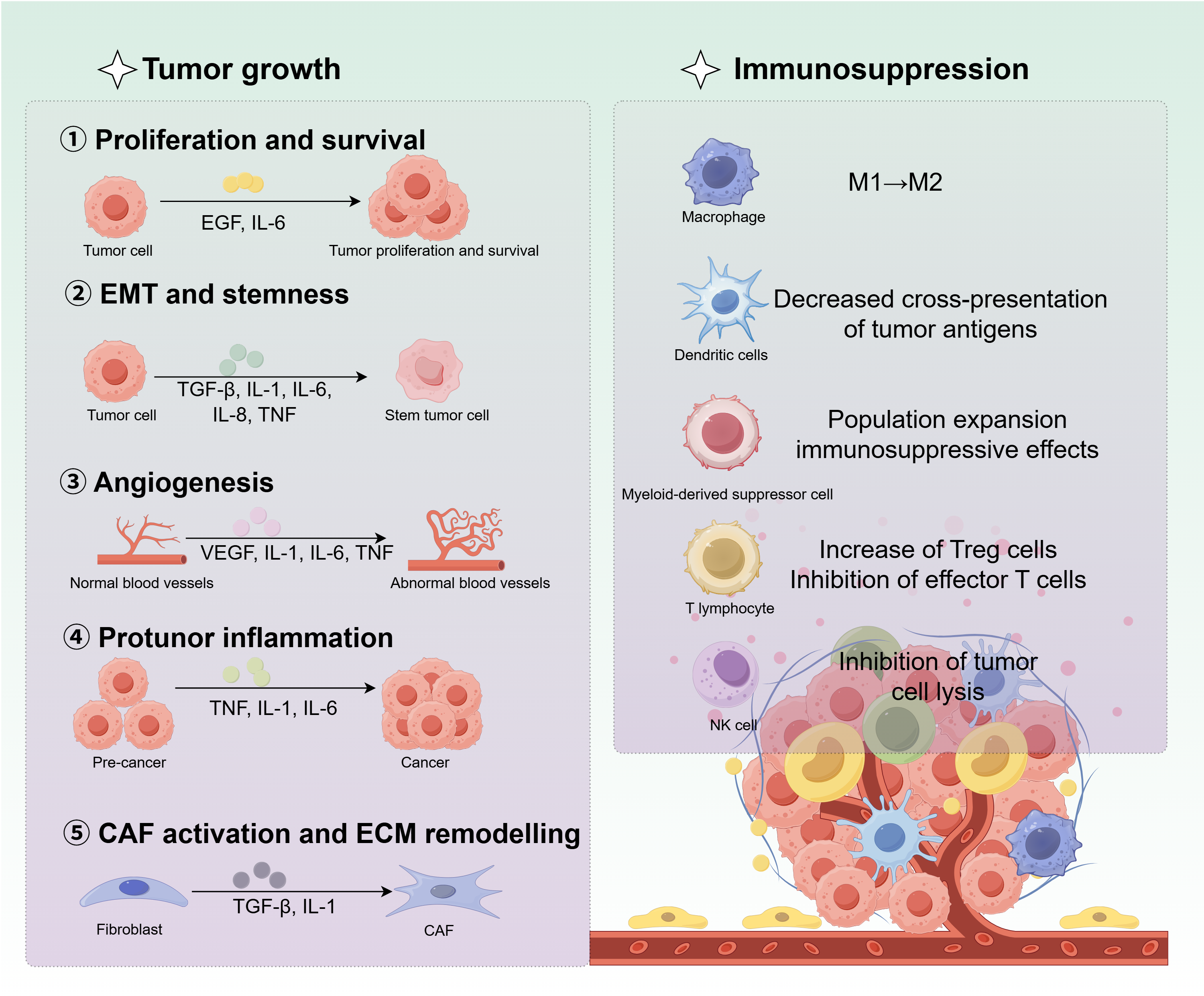

Supplement: Supplementary Figure 1 — Mechanisms of cytokines promoting cancer progression. Cytokines can promote the proliferation and survival of tumor cells; facilitate epithelial-mesenchymal transition (EMT) and stemness maintenance of tumor cells, thereby fostering a more invasive phenotype; drive angiogenesis; participate in the activation of fibroblasts into cancer-associated fibroblasts (CAFs) and the remodeling of the extracellular matrix (ECM), which in turn promote tumor immune evasion and therapeutic resistance; and pro-inflammatory cytokines induce dysregulated inflammation to support tumor initiation and progression. Additionally, cytokines act on immune cells within the tumor microenvironment to form an immunosuppressive tumor microenvironment, helping tumors evade immune surveillance and further proliferate. [file Image1.tiff]
